# Relationship between Wine Consumption, Diet and Microbiome Modulation in Alzheimer’s Disease

**DOI:** 10.3390/nu12103082

**Published:** 2020-10-10

**Authors:** M. Victoria Moreno-Arribas, Begoña Bartolomé, José L. Peñalvo, Patricia Pérez-Matute, Maria José Motilva

**Affiliations:** 1 Institute of Food Science Research (CIAL), CSIC-UAM, c/Nicolás Cabrera 9, Campus de Cantoblanco, 28049 Madrid, Spain; b.bartolome@csic.es; 2 Institute of Tropical Medicine, Unit Noncommunicable Diseases, Natl Str 155, B-2000 Antwerp, Belgium; jpenalvo@itg.be; 3 Biomedical Research Center of La Rioja (CIBIR) c/Piqueras 98, 26006 Logroño, Spain; cpperez@riojasalud.es; 4 Institute of Grapevine and Wine Sciences (ICVV), CSIC-University of La Rioja-Government of La Rioja, Autovía del Camino de Santiago LO-20 Exit 13, 26007 Logroño, Spain; motilva@icvv.es

**Keywords:** Alzheimer’s disease, diet, wine, microbiome modulation, oral and gut microbiota, polyphenol metabolites

## Abstract

Alzheimer’s disease (AD) is a progressive neurodegenerative disorder leading to the most common form of dementia in elderly people. Modifiable dietary and lifestyle factors could either accelerate or ameliorate the aging process and the risk of developing AD and other age-related morbidities. Emerging evidence also reports a potential link between oral and gut microbiota alterations and AD. Dietary polyphenols, in particular wine polyphenols, are a major diver of oral and gut microbiota composition and function. Consequently, wine polyphenols health effects, mediated as a function of the individual’s oral and gut microbiome are considered one of the recent greatest challenges in the field of neurodegenerative diseases as a promising strategy to prevent or slow down AD progression. This review highlights current knowledge on the link of oral and intestinal microbiome and the interaction between wine polyphenols and microbiota in the context of AD. Furthermore, the extent to which mechanisms bacteria and polyphenols and its microbial metabolites exert their action on communication pathways between the brain and the microbiota, as well as the impact of the molecular mediators to these interactions on AD patients, are described.

## 1. Introduction

The healthy human brain contains eighty-six thousand million neurons and many more glial cells. Each neuron can contact thousands or even tens of thousands of others. They send messages between different parts of the brain, and from the brain to the muscles and organs of the body. Alzheimer’s disease (AD) disrupts this communication among neurons, resulting in loss of function and cell death [1]. Accumulating evidence indicates that the underlying neuropathological mechanisms associated with the onset of AD begin as much as 20 years before symptoms arise, with gradual changes in the brain that pass unnoticed to the person affected before the emergence of mild cognitive impairment. As a progressive condition, AD typically disrupts neuronal connections in parts of the brain involved in memory, including the entorhinal cortex and hippocampus. It later affects areas in the cerebral cortex responsible for language, reasoning, and social behavior. Eventually, many other areas of the brain are damaged. Over time, a person with Alzheimer’s gradually loses his or her ability to live and function independently [2].

All types of dementias, including AD, have in common a series of stereotyped changes including brain inflammation, loss of synaptic plasticity, and many biochemical and metabolic changes that eventually lead to neuronal death. AD is diagnosed by the presence of lesions in the brain often referred as senile plaques, mainly composed of amyloid-β (Aβ) peptides, and neurofibrillary tangles, also as the result of protein accumulation [3]. In AD, abnormal chemical changes cause tau protein to detach from microtubules and stick to other tau molecules, forming threads that eventually join to form tangles inside neurons. These tangles block the neuron’s transport system, which harms the synaptic communication between neurons. Chronic inflammation may be caused by the buildup of glial cells normally meant to help keep the brain free of debris. One type of glial cell, microglia, engulfs and destroys waste and toxins in a healthy brain. In Alzheimer’s, microglia fail to clear away waste, debris, and protein collections, including Aβ plaques. Astrocytes -another type of glial cell- are signaled to help clear the buildup of plaques and other cellular debris left behind. Mostly, astrocytes are drawn to inflammatory sites and once activated, they become hypertrophic and contribute to inflammatory processes by releasing pro-inflammatory cytokines. Activated astrocytes also produce apolipoprotein E (APOE) which can participate in fibrillation Aβ. Over a period of months or years, the cycle of continuous release of pro-inflammatory cytokines and amyloidosis exacerbates neuronal damage. In addition, vascular problems may lead to reduced blood flow and oxygen to the brain, as well as a breakdown of the blood-brain barrier (BBB), which usually protects the brain from harmful agents while allowing glucose and other necessary factors. In a person with Alzheimer’s, a faulty BBB prevents glucose from reaching the brain and prevents the clearing away of toxic Aβ and tau proteins. This results in inflammation, which adds to vascular problems in the brain [4].

Increasing age is the most important non-modifiable risk factor for dementias and, in particular, for AD (accounting or 50–70% of total dementia cases) [5]. As life expectancy increases and demographic ageing occurs in populations around the world, the burden of dementia is expected to increase drastically. According to the World Health Organization (WHO) [6], dementia including AD and vascular dementia, affected 47 million of people globally (approximately 5% of the world population of advanced age) in 2015. This figure is projected to increase to 75 million in 2030 and 132 million in 2050, with 9.9 million new cases every year. A sizeable part of this increase will happen in low- and middle-income countries, with 68% of all AD patients estimated to live in these areas in 2050 [6]. 

In high-income countries, such as the USA, the ‘National Plan to Address Alzheimer’s Disease’ has been running for years as a national strategy, including prevention trials, and infrastructure supporting open access to big data, in order to address the scientific needs for prevention and treatment of AD by 2025 [7]. As a result of these committed policies, new trends are emerging, speeding the development of effective interpretations of disease classification and/or preventive and therapeutic strategies. For example, it is becoming clear that AD is more than a simply disorder of cognitive symptoms, and neuropsychiatric symptoms are both highly prevalent and distressing to patients and caregivers alike [8]. 

**Figure 1:**
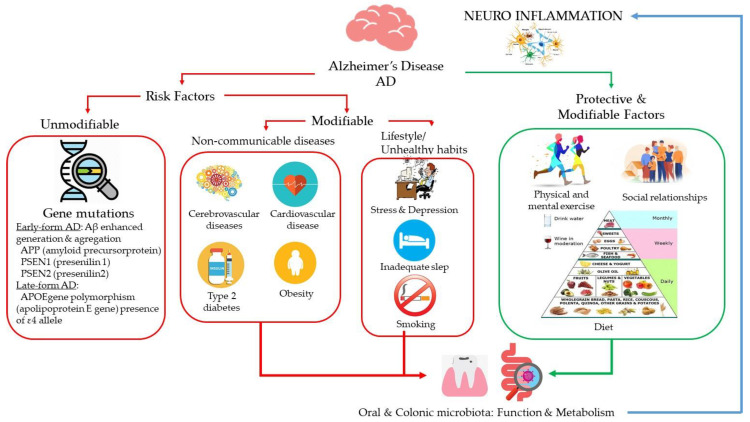
Genetic, environmental, and lifestyle factors known to determine brain functions and Alzheimer’s disease (AD) onset. APOE: Apolipoprotein E.

The causes of AD are not completely understood, but it is believed they include a combination of genetic, environmental, and lifestyle factors (Figure 1). Since it is unknown how different predisposing genes can lead to disease (or protect against it) nor how they interact with different environmental situations, several studies attempt to learn more about gene-gene and gene-environment interactions. Early AD usually occurs due to mutations in genes APP (amyloid precursor protein), PSEN1 (presenilin 1), and PSEN2 (presenilin 2), which enhanced generation and aggregation of Aβ [9]. Whereas late-form AD is mainly associated with a polymorphism in APOE gene (Apolipoprotein E gene), especially the presence of ε4 allele [10]. Besides, genetic analyses have demonstrated that, individual differences of AD could be resulted from multiple genes and their variants, exerting various biological functions in coordination to enhance the risk of the disease [11,12]. Nevertheless, the understanding of key candidate genes and pathways related with to the pathogenesis of AD is still incomplete and, therefore, does not allow any intervention until now.

Other than the above mentioned unmodifiable factors, a number of acquired factors have been identified as probable risk factors that could lead to AD (Figure 1). Among those risk factors are cerebrovascular diseases (hemorrhagic infarcts, small and large ischemic cortical infarcts, vasculopathies, and changes in cerebral white matter), coronary heart disease, Type 2 diabetes, hypertension, obesity and dyslipidemia (elevated cholesterol level) [13]. In addition, some lifestyle factors as stress, depression, inadequate sleep and unhealthy habits as smoking may affect the risk of developing AD [14]. In line with this, addressing modifiable risk factors are considered to be the most promising strategy to prevent AD and/or hinder its progress. Among the diet/lifestyle factors that may prevent or slow age-related neurodegenerative diseases, epidemiological studies have suggested that a moderate consumption of wine (particularly red wine), especially as part of a holistic Mediterranean diet (MD), correlates with better cognition in elderly populations [15,16], highlighting the conclusions of a review of the Working Group on Nutrition and Mental Performance, under the auspices of the International Life Sciences Institute Europe (ILSI Europe) [17]. Diet is a major driver of gut microbiota composition and function [18,19]. Consequently, dietary-health effects could theoretically be mediated and optimized as a function of individual’s gut microbiome and its response to the diet. The microbiota that colonizes the gastrointestinal tract and its collective genome (microbiome), orchestrates an array of bodily and brain functions (metabolic, immune, endocrine, neural, etc.) through interactions with the host and the environment (diets, antibiotics, stress, etc.) contributing to human physiology and health maintenance [20]. Unlike the host’s genome, the gut microbiome shows flexibility, representing a preventive/therapeutic target. Emerging evidence also suggests that a plethora of metabolites resulting from diet-host-microbe interactions are awaiting to be discovered and could represent a rich source of new bioactive molecules related to our health trajectory to be exploited for preventive purposes [21,22]. In relation to wine, its phenolic fraction has been suggested to modulate gut microbiota inducing prebiotic-like effects on bacteria (through the stimulation of the growth of beneficial bacteria and the inhibition of pathogen bacteria) [23]. On the other hand, intestinal bacteria metabolize wine polyphenols into specific bioavailable metabolites. Actually, the beneficial actions reported for wine have been attributed to these phenolic microbial-derived metabolites rather to the initial precursors containing in wine [24,25,26]. 

Regarding AD, the influence of the gut microbiome in the bidirectional crosstalk between gut and the brain, known as the gut-brain axis, constitutes a research field of growing interest [27]. Particularly, emerging evidence reports putative effects of the gut microbiome- polyphenol interaction on brain function and cognitive decline [28,29,30]. On the other hand, polyphenols interaction with the human body starts in the mouth when the food is ingested. In fact, the alimentary tract is a continuous tube from the oral cavity to the anus, and the bacterial composition of the entire tract, including oral microbiota, is also likely to be influenced by diet/wine components. Evidence linking oral bacteria to AD is also increasing in the last years [31,32,33].

Considering the limited efficacy of current therapies, medical or psychological, for neurologic disorders (such as AD), the discovery of new mediators and moderators of these disorders, such as the oral and gut microbiome as well as the interactions with the diet and the lifestyle may open new diagnostic, preventive and nutritional and pharmaceutical therapeutic avenues for mental conditions and, particularly, for AD.

In this paper, we have summarized the current knowledge regarding the influence of diet (in particular, moderate wine consumption) on human microbiota and its potential impact on AD. Three main sections structure the content in a sequential and concatenated way. Section 2 recompiles what is known about the impact of lifestyle and dietary patterns in AD, with special focus on moderate wine consumption as part of the Mediterranean diet, and specifically on polyphenols as wine components. The gut microbiota as the main determinant of the impact of diet on health, and other important microenvironments of the gastrointestinal tract related to AD as the oral microbiota, are described in Section 3. Last, Section 4 addresses the microbiome modulation in AD, and the contribution of wine polyphenols and/or their microbial metabolites to it. A final section tries to highlight main conclusions and future directions.

## 2. Lifestyle and Dietary Patterns and Alzheimer’s Disease

As with other chronic diseases related to aging, the need of preventive measures has become apparent, and most current approaches to tackle cognitive decline (the earliest manifestation of dementia) rely on lifestyle changes. Although both genetic and lifestyle factors play a role in determining individual risk of AD and dementia in general [34], recent large population-based cohort studies have shown that a favorable lifestyle is associated with a lower dementia risk among participants with high genetic risk, suggesting that adherence to a healthy lifestyle may offset genetic risk for dementia [35]. There is indeed mounting evidence indicating that overall healthy, non-smoker, physically active individuals who adhere to a healthy diet have a lower risk of dementia [36,37,38]. 

Smoking has been associated with an increased risk of all-cause dementia [relative risk 1.30, 95% confidence interval: 1.18–1.45], AD [relative risk 1.40, 95% confidence interval: 1.13–1.73] and vascular dementia [relative risk 1.38, 95% confidence interval 1.15–1.66] [39]. Albeit no effect modification was found for gender or race for all-cause dementia, the significantly increased risk of AD of current smokers was mostly observed among APOE ε4 non-carriers [40]. Physical inactivity has been found to explain approximately 13% of AD cases worldwide [41], and, indeed, recent meta-analyses reported improvements in global cognitive ability and positive albeit small effect on memory in people with mild cognitive impairment [42]. Lifestyle is strongly linked to metabolic risk factors, such as excess weight. Either as a proxy for unhealthy lifestyle or potentially having a role in the pathophysiology of dementia, overweight and obesity are markedly associated with increased risk of dementia [43]. Obesity, and particularly central obesity (larger waist circumference), have been associated with dementia incidence independent of demographics, lifestyle behaviors, APOE-ε4, hypertension and diabetes [44].

### 2.1. Diet

Diet is a modifiable environmental factor that has been associated with many non-communicable diseases with connections to dementia, such as diabetes and cardiovascular disease (Figure 1). A large body of scientific evidence, mostly from observational studies, suggests a direct role for lifelong nutrition on clinical measures of cognitive status in older adults [45]. However, it is not clear whether diet-induced effects on neuro-cognition are mediated directly by neuro-inflammatory processes and/or via other immune mechanisms in vivo. An increasing body of evidence suggests that peripheral inflammation and alterations to the gut microbiome can amplify neuro-inflammation and accelerate neurodegeneration [46] and these external factors can also be influenced by diet [45].

Owing to the complex biological interactions between different components of the diet, it has been proposed that the use of a whole-diet approach, through the study of dietary patterns rather than individual nutrients or food groups, might help to understand the role of diet in AD and related dementia [47]. At present, evidence of an association between diet and cognitive outcomes is somehow stronger for healthy dietary patterns, such as the Mediterranean-type diet, than for individual nutrients and food groups, possibly because of the cumulative beneficial effects of the many ingredients in these diets [48]. The ten principles of the MD are (https://dietamediterranea.com/en/fundacion): (i) use olive oil as your main fat source; (ii) eat plenty of plant products such as fruits, vegetables, legumes and nuts; (iii) bread and other grain products (pasta, rice, and whole grains) should be a part of the everyday diet; (iv) fresh and locally low-processed products are preferred; (v) consume dairy products on a daily basis, mainly yogurt and cheese; (vi) red meat should be consumed in moderation and if possible as a part of stews and other recipes; (vii) consume fish abundantly and eggs in moderation; (viii) fresh fruit should be your everyday dessert and, sweets, cakes and dairy desserts should be consumed only on occasion; (ix) water is the beverage of choice, wine should be taken in moderations and with meals; and (x) be physically active every day.

In this line, the MIND (Mediterranean–Dietary Approaches to Stop Hypertension (DASH) Intervention for Neurodegenerative Delay) diet incorporates the DASH (Dietary Approaches to Stop Hypertension) diet, which has been shown to lower high blood pressure [49], a risk factor for AD. This diet has been found to slow cognitive decline and to reduce the incidence of AD [50,51]. A meta-analysis of 43 prospective cohorts found a protective effect of MD [Relative Risk (RR) 0.69, 95% Confidence Interval (CI): 0.57–0.84]. Diets with a suboptimal fat composition, e.g., predominant saturated and trans fat intake, have been also studied in relation to the risk of dementia, including AD [52,53]. Although the biochemical mechanism is not yet fully understood, cholesterol appears to be an important risk factors of AD, being involved in both the generation and deposition of Aβ, through its interaction with the APOE-ε4 isoform, the principal cholesterol transport in the brain and the main risk factor for AD [54]. Recent metabolomic studies on brain tissue found that the unsaturated fatty acid metabolism is significantly dysregulated in the brain of patients with varying degrees of AD [55]. Other nutrients, such as homocysteine-related vitamins have also been reported to have a role in the pathogenesis of AD [56]. Indeed, in meta-analysis of cohort data, unsaturated fatty acids [RR 0.84, 95%CI: 0.74–0.95], vitamin B [RR: 0.72, 95%CI: 0.54–0.96], as well as antioxidants [RR 0.87, 95%CI: 0.77–0.98], have been associated with lower risk of dementia [39]. Other dietary factors such as aluminum (RR 2.24, 95%CI: 1.49–3.37], and low levels of vitamin D (RR 1.52, 95% CI: 1.17–1.98], have been associated with increased risks [39].

### 2.2. Alcohol

While excessive alcohol use is an established risk factor for multiple chronic diseases [57], the link between moderate alcohol consumption and degenerative conditions, such as dementia, remains uncertain. A number of studies have described a milder cognitive decline or even a decreased risk of dementia among those individuals reporting a moderate alcohol consumption compared to abstainers [58,59,60,61]. A recent meta-analysis of prospective studies involving 73,300 participants and 4586 cases for all-cause dementia reported a nonlinear association between alcohol intake and risk of dementia. The alcohol dose associated with the lowest risk of dementia was confined to a maximum of 12.5 g/day, with the lowest risk observed for 6 g/day (approximately half drink depending on the type of alcohol) while excessive drinking (≥38 g/day) elevated the risk substantially [62]. The potential role of APOE genotype in modulating the risk of dementia with increased alcohol consumption remains unclear with studies pointing in both directions [61,63].

### 2.3. Wine

Although the long-term high consumption of alcoholic beverages has been associated with an increased prevalence of cancer, cardiovascular diseases, liver cirrhosis, dementia and depression [64,65], moderate and regular red wine consumption has been shown to have a protective association, whereas beer and spirits have been reported as either not related or poorly related to cognitive outcomes [66,67,68,69]. Confidence in these associations is restrained because no clinical trials exploring the cognitive benefits of wine/alcoholic beverages have been completed. 

The first results suggesting a protective effect of moderate consumption of red wine in AD were published in the late 90s using data from a prospective cohort of elder participants in the Bordeaux area [70]. Later, additional longitudinal studies have reported similar results and confirmed that the association is mostly observed for red wine [65,66]. There is no internationally accepted definition for “drinking in moderation”, although in some countries like Australia, wine moderate drinking is defined as approximately two standard units per day for men and women, considering that a standard unit equates to around 100 mL of wine at 13% *v/v* [64]. 

In contrast to other alcoholic beverages, such as spirits, for which increased risks have been reported [71], the protective associations reported for wine may be explained by components other than ethanol. Wine is considered a dietary source of phytochemicals, and, in particular, red wine is rich in a great variety of polyphenolic compounds with potential neuroprotective activities. Red wines have a substantially higher total phenolic content than white wines (an average of 2 g/L vs. 200 mg/L). Wine polyphenols are divided into two categories: the flavonoids and non-flavonoids. The flavonoids account for most of the polyphenolic components in red wine (>85%, ≥1 g/L) and comprise of compounds such as anthocyanins (e.g., malvidin-3-O-glucoside), flavan-3-ols as monomeric [e.g., (+)-catechin], oligomeric (e.g., procyanidin B2) and polymeric forms, and flavonols (e.g., quercetin-3-O-glucoside). The non-flavonoid portion includes hydroxybenzoic acids (e.g., gallic acid), hydroxycinnamic acids (e.g., caffeic acid), hydrolysable tannins, and most importantly, resveratrol, a stilbene derivative [72]. 

## 3. Oral and Gut Microbiota in Alzheimer’s Disease

Millions of human microbiomes have been sequenced [73]. Achieving this figure has been possible thanks to the creation of large public consortia such as the ‘Human Microbiome Project’ in the USA, the ‘MetaHits’ project between China and EU, the ‘ElderMet’ project in Ireland, or the initiatives for the microbiomes and metagenomes sequencing of the Canadian and Japanese governments. Companies specializing in this type of analysis have also emerged in recent years. The work carried out by all of them has allowed us to handle a vast amount of information that allows us to conclude that the human microbiome is not homogeneously distributed in our body. There are organs with a greater number of microorganisms such as the digestive tract, but there is also other complex environment such as the mouth/oral microbiome, which is in continuous communication with the external environment and, therefore, constitutes the main entry for many microorganisms to our body with relevant consequences for human health. Up to now, approximately 700 taxa mostly belonging to bacteria taxonomic group have been identified in the human oral cavity. The Human Oral Microbiome Database (HOMD) website [74] contains detailed information about the characteristics, genomic and phylogenetic information of oral bacteria. The major oral bacteria phyla comprise Firmicutes (*Gemella, Granulicatella, Streptococcus* and *Veillonella* genera), Bacteroidetes (strongly represented by *Prevotella*), Proteobacteria (*Neisseria* and *Haemophilus* genera), Actinobacteria (*Corynebacterium, Rothia*, and *Actinomyces* genera), and Fusobacteria (genus *Fusobacterium*). The role of these oral microorganisms includes digestion of food, resistance against pathogens, maintenance of homeostasis, and the modulation of the immune system, contributing to oral and general well-being. However, they are also responsible for a variety of oral diseases [75,76]. Moreover, from an ecological perspective, it is important to emphasize that the oral microbiota is a reservoir that can transfer microbial strains to other parts of the body, such as the digestive system.

The human gut microbiome is mainly constituted by representatives of bacteria, but also include archaea, lower and higher eukarya and viruses. The intestinal ecosystem brings together the best environmental parameters for bacterial development. That is why it is the area of our body where the highest density of microorganisms with great complexity are housed; more than 1000 microbial species and all of these bacteria encode a microbial gene pool, exceeding the size of the human genome, known as the microbiome. These inhabitants of the human body are separated in different phyla. Among them, adult human gut microbiota is mainly represented by the phyla Firmicutes (including *Clostridium, Enterococcus, Lactobacillus*, and *Ruminococcus* genera), Bacteroidetes (including *Bacteroides* and *Prevotella* genera) and Actinobacteria, which represent approximately 90% of the microbiota. Other subdominant or minor phyla include Proteobacteria, Fusobacteria, and Verrucomicrobia [77,78]. The main known function of gut microbiota is to help in the harvesting of nutrients and energy from our diet; moreover, other functions such as the development of a host’s immune system, brain, and behavior; protective role against pathogens; and is a factory of bioactive compounds [79,80]. 

In Table 1, the most relevant studies on the action of oral and intestinal microbiota in connection to AD are presented. Evidence linking oral bacteria to AD is accumulating in the last years, suggesting that certain bacterial phyla, oral anaerobes, are closely associated with AD, since they were not as heavily represented in oral samples from non-AD patients [30,31,81]. Oral microbiota in oral cavity and saliva form biofilms on tooth surfaces (i.e., dental plaque) and the tongue (i.e., tongue coating). These biofilms are involved in the development of the most common oral diseases including caries, periodontitis and halitosis [82]. Dysbiotic oral microbiota (imbalance in microbiota composition) is the main cause of these pathologies; indeed, old age induces changes in oral microbiota and can exacerbate inflammation. In particular, inmunosenescence results in an increased anaerobic bacterial load and virus as cell-mediated and humoral response vane [30]. Recent scientific evidences suggest that pathogenic bacteria present in dental plaque could enter into the bloodstream and their metabolites/derived molecules pass through BBB, after deteriorating its permeability, and reach the brain, where they could promote an increase in the levels of inflammatory cytokines, and cell and vascular adhesion molecules [32]. This is consistent with evidence of endotoxin lipopolysaccharide (LPS), from the oral anaerobe *Porphyromonas gingivalis* in the brains of AD patients but not in healthy individuals. *P. gingivalis,* a keystone pathogen in chronic periodontitis, and their major virulent factors, toxic gingipains, were higher in brain of AD patients compared to controls [31,32]. Serum antibodies for periodontal disease bacteria (Immunoglobulin G levels) have been found at an elevated level in AD patients, compared to control. These antibodies play a crucial role in the progression of AD [83]. Moreover, *P. gingivalis* gingipains—lysine or arginine specific cysteine proteases (kpg, rpgA and rpgB)—are able to invade and colonize the host cells, and disrupt host immune system manipulating cytokine networks, involved in bacteria adhesion, and inactivating protease inhibitors. Subsequently, *P. gingivalis* was able to initiate microglial cell activation and promote the synthesis of innate immune inflammatory proteins [84]. Furthermore, the first evidence of a periodontal bacterial infection resulting in injury of the hippocampus, thereby increasing BBB permeability, has been recently reported. Consecutive *P. gingivalis* infections increased earlier occurrence of age-related granules in APOE−/− mice following inflammation-mediated tissue injury, accompanied by loss of cerebral BBB integrity [85]. 

Going one step further, other studies showed the potential of the detection of periodontal pathogens as an AD predictive tool. Biological resilient adults (*n* = 158), all cognitively normal, were studied in a longitudinal neurological program at the University of Kentucky. Raised baseline antibody levels, specific for the oral anaerobes *F. nucleatum* and *Prevotella intermedia*, correlated with cognitive deficits in subjects 10 years later [83]. These results were further supported by a six month observational cohort study that enrolled 60 community welling participants with mild to moderate AD showing that periodontitis is associated with an increase in cognitive decline in AD, independent to baseline cognitive state, which may be mediated through effects on systemic inflammation [86]. 

Dysbiosis has been associated in the literature with several health dysfunctions, including colitis, obesity, irritable bowel syndrome, allergies, cancer, and also brain diseases. In recent years, numerous publications on the relation between AD and the gut microbiota have also become available (Table 1)**.** Metagenomic techniques have proved different taxonomic levels in the microbiota composition of the Alzheimer’s patients compared to healthy controls or elderly without dementia [87,88]. However, until now, few studies supported in vivo a causative effect between gut dysbiosis and neurobehavioral abnormalities. The study of Kelly and co-workers showed the potential to transfer depressive-like behavioral and physiological traits via the microbiota in rodents [89]. Most recently, gut microbiota–metabolomics signatures preceding dementia were tested used the triple transgenic (3xtg) mice model, reported coherent associations between microbiota profile and cognitive impairments [90]. 

The evidence on the role of the gut microbiota on AD includes direct actions of bacteria as well as indirect actions or aging-related processes [91,92]. In light of this, recent studies have shown that bacteria are involved in the pathology of AD by altering the permeability of the BBB and, thereby, facilitating an overproduction and aggregation of Aβ. Once generated, the latter hypothetically triggers a systemic inflammatory response, which compromises complex brain functions, such as learning and memory [93]. Furthermore, feces of patients with brain amyloidosis and cognitive impairment contain more pro-inflammatory gut bacteria and blood more pro-inflammatory cytokines compared to patients with cognitive impairment without amyloidosis or controls. In addition, less anti-inflammatory bacteria and cytokines are observed [94]. Clinical studies have shown that, in cognitively impaired elderly patients with brain amyloidosis, there is lower abundance in the gut of *Eubacterimum rectale* and *Bacteroides fragilis*, two bacterial species that have an anti-inflammatory activity, *versus* a greater amount of pro-inflammatory genera such as *Escherichia/Shigella* [95]. Other studies have suggested less abundance of butyrate-producing species: *Butyrivibrio* (*B. hungatei* and *B. proteoclasticus*), *Eubacterium* (*E. eigens, E. hallii* and *E. rectale*) and *Clostridium* sp SY8519, *R.hominis* and *F.prausnitzzi* and greater abundance of *O. splanchnicus, Odoribacter sp, K. pneumoniae, B. fragilis, E. lenta* and *Desulfovibrio* genus (*D. fairfieldensis*) [96]. In many of these relationships, the role of modulation exerted by the microbiota on the inflammatory state of patients and neuro-inflammation has an important weight, which needs to be studied with a preponderant role of those bacteria capable of producing short-chain fatty acids (SCFA). Bacteria, such as those from the *Clostridium*, *Eubacterium*, and *Butyrivibrio* genera, are able to produce butyrate in the gut lumen at mM levels [97]. Butyrate is also utilized by microbiota and serves as the primary energy source of colonocytes making this a vital and mutually beneficial relationship. Interestingly, preliminary animal and human studies indicate that the colonic microbiota may be affected by oral bacteria, such as *P. gingivalis*, leading to dysbiosis [98]. In particular, long-term oral ingestion of *P. gingivalis*, similarly to periodontitis, may influence intestinal dysbiosis. Apart from *P. gingivalis*, other periodontopathogens including *A. actinomycetemcomitans*, can also disseminate to the colon [99]. Therefore, this oral–colon link may constitute another route for oral bacteria-mediated systemic inflammatory responses, which needs to be properly explored. 

As far as the intestinal level is concerned, studies have also shown that gut microbiota regulates the function of different body systems through interactions between the gut and distant organs, including the so-called ‘gut-brain axis’, via immune, endocrine and neural routes that are not fully understood [19,100]. Pro-inflammatory cytokines as well as microbial stimuli LPS, lipoteichoic acids, etc.) of non-dietary nature may cause damage in the intestinal issue but also in the brain. LPS causes the activation of the well-recognized signaling cascade of the nuclear factor-kB (NF-kB) which when permanently hyperactivated leads to chronic intestinal inflammation and dysbiosis. Both processes impact the brain function with pro-inflammatory cytokines able to dysregulate neurotransmission and alter behavior. Moreover, LPS can be recognized by microglia and astrocytes, affecting the regulation of neurogenesis and synapsis, triggering neuro-inflammation [26,101]. Pro-inflammatory cytokines can also contribute to tight junction disruption leading to bacterial translocation. This is reflected in increased IgA and IgM levels against Gram-negative bacteria, including *Hafnia alvei, Pseudomonas aeruginosa, Morganella morganii, Pseudomonas putida, Citrobacter koseri*, and *Klebsiella pneumonia* [19]. The communication between the gut and the brain can also be established due to the production of microbial products other than LPS, mainly SCFA. Butyrate and propionate were able in vitro to reduce the permeability of the BBB induced by bacterial LPS exposure [102].

Finally, preliminary literature suggests that fecal microbiota transplantation, considered currently, as the most effective gut microbiota intervention for recurrent *Clostridioides difficile* infections, may be a promising treatment option for several neurological dysfunctions [92]. In fact, some studies suggested a beneficial effect of fecal microbiota transplantation from young healthy donors, but evidence was restricted to a limited number of animal model studies. Although no published studies in humans with AD were found, the ClinicalTrials.gov database [103] showed several ongoing trials with fecal transplantation in AD patients.

**Table 1:** Studies associated with alteration of the oral and intestinal microbiota and Alzheimer’s disease (AD).

Study	Design, Aims and Details	Digestive Tract Compartment	Key Findings
Exploring the association between AD, Oral Health, Microbial Endocrinology and Nutrition [104]	Scientific literature review	Oral	Healthy diet based interventions together with improved life style/behavioral changes may reduce and/or delay the incidence of AD.
The Microbiome and Disease: Reviewing the Links between the Oral Microbiome, Aging, and Alzheimer’s Disease [31]	Scientific literature review	Oral	Epidemiological and experimental evidence links oral bacteria found in brains and oral bacteria and tumor necrosis factor in blood in AD. Combining human genetic factors with microbiome composition greatly improves the predictive capacity for assessing disease risk.
The Possible Causal Link of Periodontitis to Neuropsychiatric Disorders: More Than Psychosocial Mechanisms [105]	Scientific literature review	Oral	Periodontal bacteria/bacterial molecules can directly invade the brain either through the blood stream or via cranial nerves. In periodontitis, a periodontal pocket is filled with periodontal bacteria/bacterial molecules that form biofilms. Oral bacteria are capable of invading an intact pocket epithelium, and gain access to the circulation.
Oral microbiota and AD: Do all roads lead to Rome? [81]	Scientific literature review	Oral	Oral microbiota produces inflammatory mediators able to migrate into the bloodstream and affect distant tissues and organs, thus representing a source of neuro-inflammation.
Association between chronic periodontitis and the risk of AD: a retrospective, population-based, matched-cohort study [106]	Retrospective matched-cohort study: 9291 patients diagnosed with chronic periodontitis (1997–2004)	Oral	10-year chronic periodontitis exposure was associated with a 1.707-fold increase in the risk of developing AD.
Periodontitis and Cognitive Decline in Alzheimer’s Disease [107]	Six month observational cohort study (*n* = 60 participants with mild to moderate AD). To determine if periodontitis in AD is associated with both increased dementia severity and cognitive decline.	Oral	Periodontitis is associated with an increased systemic pro inflammatory state, and increase in cognitive decline in AD, independent to baseline cognitive state, which may be mediated through effects on systemic inflammation.
Chronic *P. gingivalis* infection accelerates the occurrence of age-related granules in APOE−/− mice brains [85]	Age-related granules in the apolipoprotein E gene knockout (APOE−/−) B6 background mice brains following chronic gingival infection with *P.gingivalis* for 24 weeks.	Oral	Periodontal bacterial infection results in injury of the hippocampus, thereby increasing blood-brain barrier permeability to toxic vascular components. Early appearance of age-related granules in APOE−/− mice following inflammation-mediated tissue injury, accompanied by loss of cerebral blood-brain barrier integrity
Determining the presence of periodontopathic virulence factors in short-term postmortem Alzheimer’s disease brain tissue [84]	Postmortem study, identifying the major periodontal disease bacteria components in brain tissue from 12 h postmostem delay (*n* = 10 AD cases for tissue from brains and 10 non-AD-related control with similar or greater postmortem interval).	Oral	LPS from periodontal bacteria can access the AD brain during life as labeling in the corresponding controls, with equivalent/longer postmortem interval. Demonstration of a known chronic oral-pathogen-related virulence factor reaching the human brains suggests and inflammatory role in the existing AD pathology
*Porphyromonas gingivalis* in Alzheimer’s disease brains: Evidence for disease causation and treatment with small-molecule inhibitors [32]	Postmortem study, identifying *P. gingivalis* DNA and gingipains, toxic proteases in AD brains	Oral	Immunohistochemical analyses using tissue microarrays showed that gingipain immunoreactivity in AD brains and that gingipain immunoreactivity significantly correlates with tau and ubiquitin loads and AD diagnosis. Using quantitative Polymerase Chain Reaction, the authors identified *P. gingivalis* DNA in the AD brains which were lysine gingipain-positive
Microbiota and Aging. A Review and Commentary [108]	Scientific literature review	Oral and Intestinal	Oral microbiota is especially important because of the opportunities for access to the brain through the olfactory nerve at the roof of the nose or through the abundant innervations of the oral cavity by the trigeminal and other cranial nerves. Communication in the gut-brain-axis is regulated by many intermediaries including diverse metabolites of the microbiota. Microbial changes have been observed in several geriatric diseases, like AD. Individuals with high frailty scores had a significant reduction on lactobacilli species when compared to non-frail individuals suggesting potential mechanisms by which the microbiota promote human health span and aging.
Secretory products of the human GI tract microbiome and their potential impact on Alzheimer’s disease (AD): detection of lipopolysaccharide (LPS) in AD hippocampus [93]	Scientific literature review	Intestinal	Presence of gastrointestinal tract microbiome-derived lipopolysaccharide (LPS) in brain lysates from the hippocampus and superior temporal lobe neocortex of AD brains. Presence of bacterial LPS hippocampal cases exhibited up to a 26-fold increase in LPS over age-matched controls.
Gut Microbiota and Their Neuroinflammatory Implications in Alzheimer’s Disease [109]	Scientific literature review	Intestinal	Impact of the microbiota of elderly people and the neuro-inflammatory roles they may have in AD, by different mechanisms: (1) role of the intestinal microbiota in homeostatic communication between the microbiota–gut–brain axis; (2) mechanisms of signal dysfunction; and (3) impact of signal dysfunction induced by the microbiota on AD
Microbiota modulation counteracts Alzheimer’s disease progression influencing neuronal proteolysis and gut hormones plasma levels [110]	Triple-transgenic mouse model of AD (3xTg-AD) mice in the early stage of AD were treated with a probiotic formulation, thereby affecting the composition of gut microbiota and its metabolites	Intestinal	Treated mice with a probiotic formulation showed partial restoration of two impaired neuronal proteolytic pathways (the ubiquitin proteasome system and autophagy). Their cognitive decline was decreased compared with controls, due to a reduction in brain damage and reduced accumulation of amyloid beta aggregates. Modulation of the microbiota induces positive effects on neuronal pathways that are able to slow down the progression of AD
Transferring the blues: depression-associated gut microbiota induces neuro-behavioral changes in the rat [89]	Thirty four patients with major depression and thirty three matched healthy controls were evaluated for the study of changes in gut microbiota, including fecal microbiota transplantation from depressed patients to microbiota-depleted rats	Intestinal	Fecal microbiota transplantation from depressed patients to microbiota-depleted rats can induce behavioral and physiological features characteristic of depression in the recipient animals, including anhedonia and anxiety-like behaviors, as well as alterations in tryptophan metabolism.
Microbiome-metabolome signatures in mice genetically prone to develop dementia, fed a normal or fatty diet [90]	To identify gut microbiota-metabolomics signatures preceding dementia in genetically prone (3xTg-AD) mice	Intestinal	3xtg mice showed brain hypometabolism typical of pre-demented stage and lacked the physiological bacterial diversity between caecum and colon seen in controls. Cluster analyses revealed distinct profiles of microbiota, and serum and fecal metabolome across groups. Elevation in *Firmicutes*-to-*Bacteroidetes* abundance, and exclusive presence of Turicibacteraceae, Christensenellaceae, Anaeroplasmataceae and Ruminococcaceae, and lack of Bifidobacteriaceae, were also observed. Metabolome analysis revealed a deficiency in unsaturated fatty acids and choline, and an overabundance in ketone bodies, lactate, amino acids, trimethylamine and trimethylamine N-oxide in 3xTg-AD mice. These metabolic alterations were correlated with high prevalence of Enterococcaceae, *Staphylococcus, Roseburia, Coprobacillus and Dorea*, and low prevalence of *Bifidobacterium*, which, in turn, related to cognitive impairment and cerebral hypometabolism
Reduction of Alzheimer’s disease Beta-amyloid pathology in the absence of gut microbiota [111]	Preclinical study: conventionally-raised transgenic APPPS1 mice aged 8-months	Intestinal	In the intestine of conventionally-raised transgenic APPPS1 mice aged 8-months, there is a significant reduction in bacteria belonging to the phyla *Firmicutes* and *Actinobacteria* with respect to an increase of *Bacteroidetes* and *Tenericutes*, supporting evidence of the role of amyloid and related bacterial accumulation in the pathogenesis of cognitive damage.
Association of brain amyloidosis with pro-inflammatory gut bacterial taxa and peripheral inflammation markers in cognitively impaired elderly [94]	Cognitively impaired patients with (*n* = 40, Amy+) and with no brain amyloidosis (*n* = 33, Amy-) and also in a group of controls (*n* = 10, no brain amyloidosis and no cognitive impairment). Studying the association of brain amyloidosis with gut microbiota taxa with pro- and anti-inflammatory activity	Intestinal	Clinical evidence of gut microbiota bacteria alterations in patients with brain amyloidosis. Abundance of the pro-inflammatory genus *Escherichia/Shigella* was significantly increased in Amyþ compared with Amy patients. Significant reduction in *E. rectale* (butyrate producer with key protective roles against inflammation) abundance in Amyþ compared with Amy_ subjects. Cognitive impairment is associated with a reduction in certain anti-inflammatory bacteria belonging to the phyla *Firmicutes* and *Bacteroidetes* compared to an increase of other pro-inflammatory bacteria of phylum *Proteobacteria.*
Gut microbiota is altered in patients with Alzheimer’s disease [112]	Fecal samples from 43 AD patients and 43 age- and gender-matched cognitively normal controls were evaluated by sequencing techniques to ascertain if the composition of gut microbiota was different between the two groups	Intestinal	Several bacteria taxa in AD patients were different from those in controls at taxonomic levels, such as Bacteroides, Actinobacteria, Ruminococcus, Lachnospiraceae, and Selenomonadales. These findings suggest that gut microbiota is altered in AD patients and may be involved in the pathogenesis of AD.
Alzheimer’s disease microbiome is associated with dysregulation of the anti-inflammatory P-glycoprotein pathway [96]	Prospective study (*n* = 108 nursing home elders, 5 months), metagenomic sequencing and in vitro T84 intestinal epithelial cell functional assays	Intestinal	Clinical parameters as well as numerous microbial taxa and functional genes act as predictors of AD dementia in comparison to elders without dementia. Less abundance of butyrate-producing species: *Butyrivibrio* (*B. hungatei* and *B. proteoclasticus*), *Eubacterium* (*E. eigens, E. hallii* and *E. rectale*) and *Clostridium* sp. SY8519, *R.hominis* and *F.prausnitzzi* in AD patients, as well as greater abundance of *O. splanchnicus, Odoribacter* sp., *K. pneumoniae, B. fragilis,* and *E. lenta* and *Desulfovibrio* genus (*D. fairfieldensis*).

## 4. Microbiome Modulation by Diet/Wine Polyphenols and Alzheimer’s Disease

Over the last 20 years, dietary polyphenolic compounds have received much attention because of their potential biological activities in many chronic diseases such as cardiovascular diseases, diabetes, obesity, and other inflammation-related diseases and lifestyle-related cancer [113,114]. The first mechanisms proposed for the action of polyphenols in the human body were based on their direct antioxidant properties; however these effects are no longer considered so relevant in vivo, since polyphenols, as a consequence of the gastrointestinal digestion process, are subjected to an intense metabolism, so that their native forms present in food do not reach the target tissues in sufficiently high concentrations to have a significant effect in terms of neutralizing free radicals [115]. In recent years, progress has been made in the identification of possible biochemical and molecular mechanisms of action of phenolic metabolites related to the modulation of endogenous antioxidant system through intracellular and intercellular signaling pathways, which depend fundamentally on ingested doses of polyphenols through food, and their effective absorption and bioavailability, which determines the concentration of phenolic metabolites that reach the target tissues [25,116,117,118].

**Figure 2:**
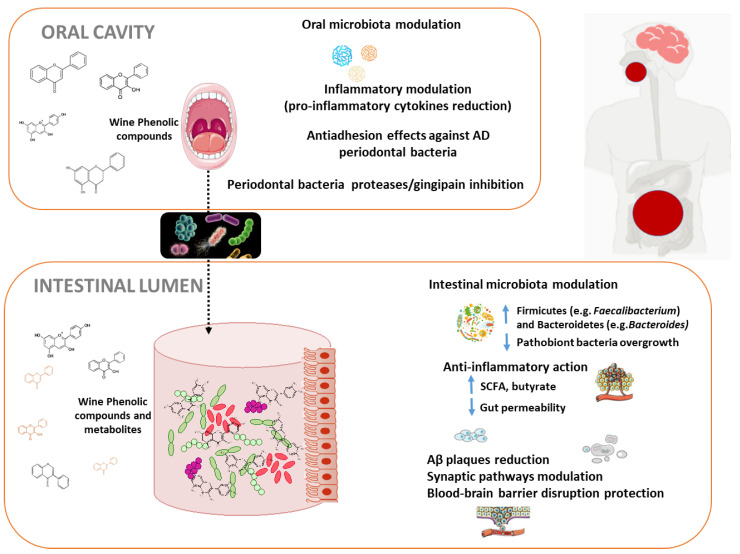
Schematic view illustrating the putative mechanisms underlying the interaction of wine polyphenols with oral and gut microbiota and protection against Alzheimer’s disease. AD: Alzheimer´s disease, SCFA: Short Chain Fatty Acids, Aβ: amyloid-β.

Red wine is one of the richest sources of polyphenols in the diet. In particular, red wine provides a unique and very diverse combination of phenolic structures including flavonols, flavan-3-ols and anthocyanins, among the flavonoid compounds, and hydroxybenzoic and hydroxycinamic acids, phenolic alcohols and stilbenes, among the non-flavonoids. Taking into account that the influence of wine polyphenols on human microbiota is becoming widely recognized, and many new studies about microbiota and AD have been reported over last few years, we want to place a special emphasis on studies that comprehensively explore, from an integral perspective, the connection between modulation of oral and intestinal microbiota by wine polyphenols, and the derived consequences in AD (Figure 2).

### 4.1. Oral Microbiota Modulation by Wine Polyphenols and Alzheimer’s Disease

Increased data link oral microbes with AD. Different hypothesis reveal how increased brain microbial burden may directly exacerbate Aβ deposition, inflammation, and AD progression. Once the oral cavity is infected, transient bacteremia of *P. gingivalis* can occur resulting in documented translocation to a variety of tissues and organs. *P. gingivalis* may access the brain and spread via different pathways including direct infection and damage to endothelial cells protecting the BBB, infection of monocytes followed by brain recruitment and/or infection and spreading through cranial nerves. Is has been suggested that after entering the brain, *P. gingivalis* spreads slowly over many years in mice from neuron to neuron along anatomically connected pathways [31]. Elderly people and AD patients’ decreased ability of oral self-care and salivary flowrates can enhance those effects. Therefore, it is of great relevance the search of inhibitors for prevent *P. gingivalis* brain colonization and neurodegeneration in AD. The dissemination of oral microorganisms to the brain is controlled by antimicrobial peptides, as part of the innate immune system. Indeed, in the last decade, several studies have explored the role of these antimicrobial peptides as potential biomarkers for AD; in particular, salivary lactoferrin discriminates between patients with mild cognitive impairment and AD from control subjects [119]. Another possible way derived from the antimicrobial properties of polyphenols, which can decrease the number of bacteria found in the biofilms of the oral cavity [120,121]. Treatments with red wine, dealcoholized wine, and oenological extracts were effective against periodontal pathogens *F. nucleatum*, *P. gingivalis* and *A. actynomycetemcomitans*, in experiments in oral subgingival biofilm models. This effect was independent of the presence of ethanol [122]. A critical step in bacterial infection is the pathogenic adhesion to host cells and an anti-adhesion therapy is an efficient way to prevent or treat bacterial infections and bacteremia. The effect of oral bacteria metabolites on AD is unknown, however in the case of *P. gingivalis* adherence, some phenolic metabolites with antimicrobial and immunomodulatory actions, such as caffeic and *p*-coumaric acids, at concentrations naturally found in wine, inhibited the adhesion of periodontal pathogens to oral cells as shown recently [122]. The combination of phenolic acids with oral probiotic *Streptococcus dentisani* resulted in a synergistic anti-adhesive effect against AD causative bacteria in oral fibroblasts with evident anti-inflammatory activity against cytokine production, all together preventing the progression of periodontal disease and promoting host-microbe homeostasis [123]. Secreted cysteine proteases, gingipains rgp and kgp, are essential for *P. gingivalis* virulence. Some polyphenols and flavonoids present in wine and tea are known to inhibit gingipain activity and interfere with biofilm formation by *P. gingivalis* in gingival cells models [124]. Resveratrol has shown significant results by targeting inflammatory and adhesive markers, and also attenuated the virulence of *P. gingivalis* by reducing the expression of virulence factor genes such as fimbriae (Type II and IV) and proteinases (kgp and rgpA) [125].

### 4.2. Intestinal Microbiota Modulation by Wine Polyphenols and Alzheimer’s Disease

After pass through the oral cavity, polyphenols suffer the action of microbiota in the gastrointestinal tract. Polyphenols can reach the colon in high proportions (90–95%), where they can be transformed into an inventory of bioavailable microbial metabolites by the resident microbiota, which are considered to be responsible for the health promoting effects that are attributed to the parent compounds. As result of this bi-directional relationship between polyphenols and the microbiome of the human gut, polyphenols can also modulate the composition of an individual’s microbiome.

Regarding wine polyphenols-gut microbiome interactions, only a few intervention studies have investigated its impact in host and mental health (see systematic review of clinical trials published between 2006 and 2018 in [126]). Interestingly, after moderate intake of red wine (4–5 weeks, 30 days of intervention depending on the study) an overall increase was observed in global microbial diversity and also in populations of Proteobacteria, Fusobacteria, Firmicutes, and Bacteroidetes at phylum level; *Enterococcus, Prevotella, Bacteroides* and *Bifidobacterium*, at genera level; and in *Blautia coccoides, Eubacterium rectale group* and *B. uniformis* and *Eggerthella* bacterial species [23,127,128,129]. As AD-associated changes in the diversity of gut microbiota imply the reduction of commensals such as bacteroides, bifidobacteria and lactobacilli [130], red wine could contribute to a beneficial gut microbiota in the context of a whole-diet approach. Lower LPS concentrations were also observed after red wine consumption for 20 days, which implies lower bacterial translocation and inflammation [127]. These beneficial effects on inflammation could be caused by either interaction with the intestinal microbiota, but also by direct anti-inflammatory effects at intestinal and systemic level [131]. Modulation of intestinal microbiota by wine polyphenols may help protect against AD, in part, by supporting the generation of select SCFAs, which interfere with the formation of toxic soluble Aβ aggregates [132]. Interestingly enough, moderate red wine consumption has also been correlated with the abundance of key health bacteria such as *Faecalibacterium prausnitzii* and *Akkermansia* sp., butyrate producers with brain anti-inflammatory properties [133,134], and positively affects the phenolic metabolic activity of various gut bacteria [135,136,137,138], and increases the SCFAs level [134,139], hence, improving host metabolism. Microbial phenolic metabolites, found in blood after wine consumption, such as 3,4-dihydroxyphenylacetic (3,4DHPA), 3-hydroxyphenylacetic (3HPA), at physiological concentrations (between 0.1–10 µM) demonstrated neuroprotective effects through on neuronal and glial kinase signaling pathways involved in the reduction of neuro-inflammation that triggers the onset of AD [27,140].

Despite these valuable findings, more robust studies with larger populations and from both taxonomic and functionality approaches are needed to confirm and complete such results in causal AD patients. Besides, an individual variability in metabolite production has been reported, depending on the specific chemical structure of the polyphenol and differences/variations in gut microbiota. Consequently, different gut microbiota-responsive phenotypes to wine polyphenol interventions have been reported [134,138]. The processes by which wine polyphenols may be absorbed and metabolized could interfere with their bioavailability and by extension with their health-promoting effects. Therefore, the identification of differential responses to red wine polyphenols intake should be also taken into consideration in future studies [135,138,141]. Important in wine health-effects in neurodegeneration is its ethanol content, a critical factor that should be taken into account in the holistic approaches on these studies. On the other hand, few studies have evaluated the effect of moderate alcohol intake in wine polyphenols bioavailability. Some studies compared the bioavailability of anthocyanins using red wine and dealcoholized red wine, and only detected the main native anthocyanin in plasma and urine with no effect of ethanol on the amount quantified. Increases in plasma of malvidin-3-O-glucoside were not significantly different after the consumption of either red wine or dealcoholized red wine [142]. Moreover, the bioavailability and biotransformation of red wine polyphenols by gut microbiota, as determined in feces after wine intake, and likewise resveratrol seems not to be affected by the alcoholic matrix of the wine [68].

The interactions and metabolic pathways of wine microbial polyphenols have been widely documented, however, most studies were carried out in in vitro colonic and cell models or animal models [24,143]. An increasingly accepted notion is that wine-related polyphenols and microbial derived metabolites exert neuroprotective and neurorescue effects via a combined ability to antagonize amyloid aggregation, suppress neuro-inflammation, modulate signaling pathways, and decrease mitochondrial dysfunction as demonstrated in-vitro [27], as well as in animal models [29,126,144,145], and in observational studies [143,146]. Polyphenols might also impact AD pathophysiology without involving active compounds reaching the brain. For example, studies in animal models of AD have shown beneficial effects without detecting specific brain alteration. Treatment with a polyphenolic extract from blueberries and grapes induced significant improvements in cognitive impairment in 3xTg-AD mice with not effects on Aβ and tau pathologies in brain [147].

As there is a crucial need for the development of new strategies capable to prevent, delay the onset or treat brain dysfunction and associated cognitive decline, different studies have focused on biological activity of individual phenolic compounds and derived microbial metabolites [114]. Interactions of some native phenolic compounds structures present in red wine (gallic acid and catechin) and seed grape (proanthocyanidins), with proteins in the brain (i.e., Aβ) have been reported as one of the protective mechanism of wine involved on AD, thereby, inhibiting amyloid fibrillation and aggregation [148]. Epicatechin and its in vivo metabolite, 3′-O-methyl epicatechin, protected human fibroblasts from oxidative-stress-induced cell death involving caspase-3 activation and neuro-inflammation [149]. Dietary polyphenolic compounds and their metabolites produced by gut microbiota showed enhanced BBB permeability, which could explain such beneficial effects in the brain [150]. Despite the accumulating evidence for free quercetin detected in the brain after oral administration, recent evidences suggest it may result from degradation of conjugated metabolite forms, since quercetin alone may not be transported along the BBB [151]. Thus, to exert their beneficial effects in target tissues and organs, polyphenols and their metabolites must to be both, bioaccessible and bioavailable. Therefore, it is imperative to understand the bioaccessible components promoting protective actions in neural cells. In this line, resveratrol, the red wine phenolic compound most intensely studied to date, exhibits strong antioxidant functions in vitro and in cell culture models and, and is thought to contribute to the cardio- and neuroprotective effects observed for moderate consumption of red wine [152]. Even though unmodified resveratrol has a modest bioavailability, there are studies pointing to strong in vivo neuroprotective properties of its metabolites [25,153]. In vivo experimental evidence also suggests that metabolites from red wines and other grape products may also benefit AD by directly modulating Aβ- as well as tau-related pathological mechanisms in the brain [154]. For example, the progress of hippocampal neurodegeneration in the brain of diabetic rats was reduced after the administration of 20 and 40 mg/kg gallic acid and p-coumaric acid, respectively [155]. Malvidin-3-O-glucoside, quercetin glucuronide, 3-hydroxybenzoic and 3-hydroxyphenylpropionic acids were able to restore synaptic plasticity in a neuroticism-like induced-situation by affecting the expression of genes involved in protein translation, as demonstrated in a model of mice primary hippocampal neurons [156]. In addition, in vivo, protocatechuic acid at a dose of 30 mg/kg prevented ischemia-induced BBB disruption and inflammatory responses mediated by microglia and astrocytes [157].

Regarding AD prevention and treatment, it is essential that bioavailability issues be addressed for neuroprotection to be relevant in a clinical study scenario. Different clinical studies have been carried out that explored the benefits of resveratrol for treating individuals having AD (see ClinicalTrials [103] for a review), but the results are not completely conclusive [158]. Resveratrol was safe and well-tolerated at doses of up to 5 g/day in humans (*n* = 20), and, even though only 1% of resveratrol reached central nervous system, AD biomarker changes were reported, including a significantly less pronounced decline in cerebrospinal fluid and plasma amyloid-beta levels (6% vs. 20%, resveratrol-treated group vs. placebo, respectively), suggesting that resveratrol had indeed engaged its target in the brain [159]. In another small clinical trial (*n* = 37), Moran et al. [160] have found no significant differences in overall cognitive function or composite cognitive domains between elderly groups of daily consumption of 150 mg resveratrol from a multi-ingredient juice for six months. Additionally, clinical trials of resveratrol were largely focused on characterizing its pharmacokinetic and metabolism or improve specific parameters, such as memory or physical performance in adults. In contrast to other wine polyphenols, resveratrol metabolites could not be monitored by conventional non-targeted metabolomic approaches (Nuclear Magnetic Resonance- and Mass Spectrometry-based), principally due to the relatively low concentrations of bioavailable resveratrol metabolites, in addition to specific technical limitations of these approaches. The pharmacokinetics of plasmatic and urinary resveratrol metabolic profiles were studied after moderate consumption of red wine by two Liquid Chromatography-Mass Spectrometry-based targeted studies [161,162]. More than twenty metabolites of resveratrol (resveratrol, piceid and sulfated metabolites) were identified including those formed by gut microbiota metabolism, showing the importance of each individual resveratrol metabolite in metabolism and the health effect of wine-derived resveratrol [161,162].

Overall, these results suggest that moderate red wine consumption may be a strategy to modulate the structure and function of the human gut microbiota, as well as for positively altering the type and amount of bacterial catabolites and bacterial-host co-metabolic products, with a potential impact on metabolic and mental health (e.g., phenolic metabolites, short chain fatty acids, neuroactive-derived compounds, etc.). Wine polyphenols could exert their effects in the context of AD through different mechanisms: (i) direct actions on the brain; (ii) through their ability to modulate gut microbiota composition and functionality and, lastly, (iii) through the actions and properties of the metabolites produced in the gut. More studies including a deep investigation of all these mechanisms are needed. Considering that metabolic changes are most likely the result of polyphenol-microbiota interactions, an integrated microbiome-metabolome study is suggested to better understand neuroprotective effects or red wine.

## 5. Conclusions and Future Directions

Giving the rapid increase in the proportion of older adults worldwide, it is of crucial importance to identify modifiable risk factors that may prevent or delay the onset of cognitive impairment and extend years of healthy life. The identification and implementation of effective dietary strategies early in the adult life, could help to improve cognition and mental health. This systematic review has tried to clarify the connections between diet, and wine in particular, and human microbiome at the frame of the AD. New challenges in the role of wine polyphenols in the prevention of functional decline and AD have been tried to be identified.

Accumulated evidence suggests that microorganisms are implicated in AD pathogenesis. In the cascade of events preceding AD, oral cavity and gastrointestinal microbiome seem to play a role and different bacteria have shown an important contribution to stimulate Aβ aggregation and neuro-inflammation. Although AD is a complex disease and microbial infection may not be the sole cause, this new paradigm may provide novel targets for the prevention and treatment of this devastating disease.

Studies in oral microbiomes seems to be critical in our understanding of AD and of how to prevent it through diet and lifestyle. The mechanism for the relationship between periodontitis and cognitive decline is still unclear but there is accumulated evidence to support a role for systemic infection and inflammation, direct entry of bacteria (particularly *P. gingivalis*) into the brain producing inflammatory mediators, so causing neuro-inflammation and possibly acting as primary agents for AD. Then strategies to prevent and/or treat periodontitis might be a possible treatment/beneficial option in countering AD progression. An adequate oral hygiene habits are the main strategy used to prevent the onset of these disorders. Dietary patterns and, in particular diets including red wine polyphenols, modulate the composition and integrity of the oral microbiota suggesting plausible benefits in the prevention of periodontal diseases. Although no published studies linking directly the modulation of oral microbes by wine polyphenols (or from other dietary sources) and AD were found, their multiple observed mechanisms of action, including antimicrobial, anti-adherent ability, inmunomodulatory effects and inhibition of virulence gene expression need to be evaluated to understand whether this orally-driven disruption will reduce *P. gingivalis* infection in the brain and slow or prevent further neurodegeneration and accumulation of pathology in AD patients. However, this research field is still in need of further in vitro and especially in vivo well-conducted studies for further support of these beneficial effects.

Current experimental evidences suggest that red wine polyphenols act in a multi-target way. Emerging in vitro and in vivo evidence reports consequences of the gut microbiome-wine polyphenol interaction on brain function and AD protection. These effects involve modulation of the composition of the gut microbiota and its functions leading to significant health consequences that are thought to be mediated through different mechanisms, including modifications in factors regulating synaptic plasticity and neural function, and the regulation of inflammatory pathways, and are driven by wine phenolic metabolites and other microbial derived metabolites (i.e., SCFA) as well as by microbial stimuli by specific intestinal bacteria. However, caution needs to be taken when interpreting the results of these studies and extrapolating them to humans, since mostly cellular and animal models have been performed. Further intervention trials are warranted to increase understanding of the impact of wine-microbiota interaction on features on mental health and AD risk. In addition, it will be important to extend these findings in larger-scale studies with adequate geographic separation. This will allow to accurately analysis of the influence of other potential confounding variables.

Cohesive research is needed through the precise and quantified integration of data from wine consumption, omics and imaging technologies, advanced models and multi-organ-on-a-chip models to mechanistic studies that offer many possibilities to address the major research challenges arising from the complexity of interactions between wine/diet polyphenols and AD preventive outcomes, as well as to advance in the development of microbiome-informed predictive tools and biomarkers of the health status and early disease detection.

## Abbreviations

AD	Alzheimer’s disease
Aβ	Amyloid-β
BBB	Blood-Brain Barrier
CI	Confidence Interval
APOE	Apolipoprotein E
DASH	Dietary Approaches to Stop Hypertension
LPS	lipopolysaccharide
MD	Mediterranean diet
MIND	Mediterranean–DASH Intervention for Neurodegenerative Delay
RR	Relative Risk
SCFA	Short-Chain Fatty Acids
3xTg-AD	Triple-transgenic mouse model of AD
WHO	World Health Organization
